# Euthanasia and Physician-Assisted Suicide in Patients With Multiple Geriatric Syndromes

**DOI:** 10.1001/jamainternmed.2020.6895

**Published:** 2020-12-07

**Authors:** Vera van den Berg, Ghislaine van Thiel, Margot Zomers, Iris Hartog, Carlo Leget, Alfred Sachs, Cuno Uiterwaal, Els van Wijngaarden

**Affiliations:** 1 Department of Care Ethics, University of Humanistic Studies, Utrecht, the Netherlands; 2 Department of Medical Humanities, Julius Center for Health Sciences and Primary Care, University Medical Center Utrecht, Utrecht, the Netherlands; 3 Department of Medical Ethics, Philosophy, and History of Medicine, Erasmus Medical Center Rotterdam, Rotterdam, the Netherlands; 4 Julius Center for Health Sciences and Primary Care, University Medical Center Utrecht, Utrecht, the Netherlands; 5 Department of Epidemiology, Julius Center for Health Sciences and Primary Care, University Medical Center Utrecht, Utrecht, the Netherlands

## Abstract

**Importance:**

The Dutch Regional Euthanasia Review Committees (RTEs) reviewed and reported an increasing number of cases of euthanasia and physician-assisted suicide (EAS) requested by older people with multiple geriatric syndromes (MGS). Knowledge of the characteristics of cases of EAS for MGS is important to facilitate societal debate and to monitor EAS practice.

**Objective:**

To examine the accumulation of patient characteristics, geriatric syndromes, and other circumstances as reported in the case summaries of the RTEs that led to unbearable suffering associated with a request for EAS and to analyze the RTEs’ assessments of these cases of EAS.

**Design, Setting, and Participants:**

A qualitative content analysis was conducted of all case summaries filed from January 1, 2013, to December 31, 2019, under the category MGS and published in a national open access database. These case summaries were selected by the RTEs from the total of 1605 reported cases of EAS in the category MGS.

**Results:**

The RTEs published 53 cases (41 [77%] female) under the category MGS. A total of 28 patients (53%) had always perceived themselves as independent, active, and socially involved. None of the patients suffered from life-threatening conditions. Multiple geriatric syndromes, such as visual impairment (34 cases [64%]), hearing loss (28 cases [53%]), pain (25 cases [47%]), and chronic tiredness (22 cases [42%]), were common. The request for EAS was often preceded by a sequence of events, especially recurrent falls (33 cases [62%]). Although physical suffering could be determined in all cases, the case descriptions found that suffering occurred on multiple dimensions, such as the loss of mobility (44 [83%]), fears (21 [40%]), dependence (23 [43%]), and social isolation (19 [36%]).

**Conclusions and Relevance:**

This qualitative study suggests that an accumulation of geriatric syndromes leading to a request for EAS is often intertwined with the social and existential dimension of suffering. This leads to a complex interplay of physical, psychological, and existential suffering that changes over time.

## Key Points

**Question:**

What are the patient characteristics and circumstances associated with the request for euthanasia and physician-assisted suicide (EAS) in cases of multiple geriatric syndromes as reported in the case summaries of the Dutch Regional Euthanasia Review Committees?

**Findings:**

In this qualitative study of 53 case summaries published by the Dutch Regional Euthanasia Review Committees, a combination of multiple geriatric syndromes, such as visual impairment, hearing loss, pain, and chronic tiredness, may have led, in most cases, to an accumulation of suffering on multiple dimensions, resulting in a request for EAS because of unbearable suffering.

**Meaning:**

This study suggests that unbearable suffering leading to a request for EAS in older persons without a life-threatening condition is often associated with a combination of medical, social, and existential issues.

## Teaser

This study from the Netherlands looks at the association between having multiple geriatric syndromes and requesting euthanasia or physician-assisted suicide.

## Introduction

Since 2002, Dutch physicians are allowed to perform euthanasia and physician-assisted suicide (EAS) when the due care criteria laid down in the Dutch Termination of Life on Request and Assisted Suicide Act (hereafter referred to as the Dutch euthanasia law) are met.^1^ One of the 6 criteria for legally permissible EAS is that “the physician must be satisfied that the patient’s suffering is unbearable, with no prospect of improvement.” (For the other criteria, see Box 1.) Each case of EAS is reported to the Dutch Regional Euthanasia Review Committees (RTEs). These committees assess and determine whether the physician acted in accordance with the due care criteria in the Dutch euthanasia law.^2^ (See the eAppendix in the Supplement for information about the RTEs’ review procedure.)

Box 1. **Criteria for Due Care in the Termination of Life on Request and Assisted Suicide (Review Procedures) Act (2002)^1^**

Requirements physician must satisfy:
  - Must be satisfied that the patient’s request is voluntary and well considered.
  - Must be satisfied that the patient’s suffering is unbearable, with no prospect of improvement.
  - Must have informed the patient about the situation and prognosis.
  - Must have come to the conclusion, together with the patient, that there is no reasonable alternative in the patient’s situation.
  - Must have consulted at least one other, independent physician, who must see the patient and give a written opinion on whether the due care criteria set out in (a) to (d) have been fulfilled.
  - Must have exercised due medical care and attention in terminating the patient’s life or assisting in suicide.

Most Dutch EAS cases involve patients who suffer unbearably because of cancer in the last phase of life. In recent years, however, an increase has been reported in EAS performed in patients with dementia, psychiatric disorders, or multiple geriatric syndromes (MGS).^3,4^ Following the Euthanasia Code 2018, a geriatric syndrome is defined as degenerative in nature, often occurring in older patients. With regard to MGS, such as sight impairment, hearing impairment, osteoporosis, osteoarthritis, balance problems, or cognitive deterioration, the Dutch RTE guidance for physicians states that these geriatric syndromes may cause unbearable suffering without the prospect of improvement “in conjunction with the patient’s medical history, life history, personality, values and stamina.”^5^^(pp 23-24)^ Although acceptance of EAS in cases of MGS is increasing in Dutch society, a majority of Dutch physicians are reluctant to grant a request for EAS on these grounds.^6^ Such requests are considered to be much more complex than those made by patients with a terminal disease, not only in ethical terms but also in legal and medical terms. For example, when does an accumulation of geriatric syndromes cause unbearable suffering without prospect of improvement? Are physicians sufficiently equipped to assess this suffering? Are these requests caused by a trend of people increasingly regarding normal decline as a disease?

This study aims to contribute to the further debate on dealing with requests for EAS from older persons with MGS. To this end, we (1) describe the patient characteristics, including the geriatric syndromes, that are associated with the request for EAS in cases of MGS; (2) explore which accumulation of syndromes and circumstances are associated with unbearable suffering in cases of MGS; and (3) attempt to gain a better understanding of the RTEs’ assessment practice.

## Methods

We studied all 53 anonymized case summaries filed under the category MGS from an open access database on the RTE website.^1^ These cases are selected by the RTEs from all 1605^2^ reported EAS cases in the category MGS from January 1, 2013, to December 31, 2019. An overview of total numbers of deaths, EAS cases, and EAS cases of MGS per year is given in Table 1.^7,8,9^ The Medical Research Ethics Committee Utrecht confirmed that our study was exempt from further ethical review, so no informed consent was required. All patient data were deidentified. This study followed the Standards for Reporting Qualitative Research (SRQR) reporting guideline. The selection of cases for publication on the website is guided by the aim to give an overview of the spectrum of cases reviewed and to contribute to the understanding of complex or controversial cases among physicians and the general public. In a meeting with a member and the chairman of the RTEs, we discussed the question of which cases are to be published in the national database. They confirmed that not only cases that address questions and dilemmas were selected but also cases representing situations that often occurred and were therefore considered common (oral communication, February 28, 2019). The length of the case summaries varies from 567 to 3130 words (approximately 2-6 pages), with a median of 1132 words. Among the more extensive case reports are the ones in which the RTE asked the physician (and sometimes also the consultant) for additional information. In these cases, the RTE had a face-to-face discussion with the physician (and consultant).

**Table 1.** Numbers of Deaths, EAS Cases, and EAS for MGS Cases per Year^a^

Year	Total No. of deaths	No. of deaths per age category	Total No. of deaths by EAS^b^	No. of deaths by EAS per age category	Total No. of EAS deaths for MGS
2013	141 245	80-89 Years of age: 49 583; ≥90 years of age: 25 229	4829	NA	251
2014	139 223	80-89 Years of age: 48 182; ≥90 years of age; 25 676	5306	NA	257
2015	147 134	80-89 Years of age: 51 283; ≥90 years of age: 27 962	5516	NA	183
2016	148 973	80-89 Years of age: 51 665; ≥90 years of age: 28 649	6091	80-89 Years of age: 1487; ≥90 years of age: 522	244
2017	150 027	80-89 Years of age: 52 397; ≥90 years of age: 29 640	6585	80-89 Years of age: 1634; ≥90 years of age: 653	293
2018	153 328	80-89 Years of age: 53 203; ≥90 years of age: 30 401	6126	80-89 Years of age: 1442; ≥90 years of age: 512	205
2019	151 793	80-89 Years of age: 52 810; ≥90 years of age: 30 089	6361	80-89 Years of age: 1628; ≥90 years of age: 504	172

Abbreviations: EAS, euthanasia and physician-assisted suicide; MGS, multiple geriatric syndromes; NA, not available.

^a^ Data are based on information from the Dutch Central Bureau of Statistics^7^ and the Dutch Regional Euthanasia Review Committees (http://www.euthanasiecommissie.nl).^8^

^b^ According to the Third Evaluation of the Euthanasia Law,^9^ 55% of the expressed requests for euthanasia are honored. It is not known how many of these cases are associated with MGS.

We conducted a directed qualitative content analysis^10^ of the cases using the analysis program ATLAS.ti, version 8.4.15 (ATLAS.ti Scientific Software Development GmbH). One author (V.v.d.B.) read all 53 documents completely to acquire an overall picture of the nature of the cases, repeatedly comparing variables of interest in light of the main research question of the study. The coding scheme was developed by 2 authors (V.v.d.B. and E.v.W.) and discussed with another (G.v.T.). All documents were coded by 1 author (V.v.d.B.) based on the predetermined codes. New findings beyond the scheme and discrepancies were discussed and resolved among 4 authors (V.v.d.B., E.v.W., G.v.T., and M.Z.) and assessed by the whole team. Given the descriptive goals of this study, the emphasis was on frequency tabulation.

## Results

The RTEs published 53 cases (41 [77%] female) under the category MGS, which were reported between 2013 and 2019. In Box 2, we first present 3 of the analyzed cases to illustrate how the combination of medical conditions and other characteristics accumulate to create a situation in which the physician became convinced that the patient was suffering unbearably without prospect of improvement.

Box 2. **Descriptions of Cases of Multiple Geriatric Syndromes^a^**

**Case 1**

A woman in the age range of 90 to 100 years had progressive vision loss and hearing impairment. She also experienced chronic pain in her legs, loss of mobility, and balance problems. A few weeks before the euthanasia and physician-assisted suicide, she fell out of bed and suffered several fractures. Since that moment, her fear of a repeated fall made it difficult for her to sleep. Because of her condition, she felt lonely and cut off from her social environment. She was not able to read or watch television and was not up to any activities anymore.

**Case 2**

A woman in her 90s had been suffering from the consequences of osteoporosis for several years. Recurrent falls caused multiple fractures. A month before her death, she underwent surgery for a hip fracture. Her recovery did not go well, and the prognosis was bleak. Loss of mobility and pain prevented her from sitting comfortably. The lack of any prospect of improvement, the loss of autonomy, being completely dependent, and the fear of losing clarity of mind together caused the unbearable suffering that was the medical grounds for euthanasia and physician-assisted suicide.

**Case 3**

A woman older than 90 years whose physical health was deteriorating was dealing with hearing loss, severe fatigue, uncontrollable headaches, restless legs, and incontinence. All her life she had been a very independent, active, and engaged person. She hated accepting help from others, and because of her worsening hearing impairment, she was not able to participate in social activities. She felt excluded from society. She feared further physical decline, with her greatest fear being forced to move to a nursing home environment.

^a^These case descriptions illustrate the most important findings of this study: (1) that falls often occur and can be a tipping point that leads to a request for euthanasia; (2) that the consequences of a single geriatric syndrome can, in some cases, be sufficient to grant a request for euthanasia; and (3) that suffering has multiple intertwined dimensions.

### Patient Characteristics

Patient characteristics and circumstances are given in Table 2. All 53 patients were 80 years of age or older and 41 (77%) were 90 years of age or older. In 28 cases (53%), it was reported that patients had always perceived themselves as independent, active, and socially involved persons.

**Table 2.** Patient Characteristics and Circumstances

Characteristic	No. (%) of cases (N = 53)
Age group, y	
80-89	12 (23)
90-100	41 (77)
Sex	
Male	12 (23)
Female	41 (77)
Geriatric syndrome^a^^,^^b^	
Visual impairment	34 (64)
(Chronic) pain	25 (47)
Hearing loss	28 (53)
(Chronic) tiredness or fatigue	22 (42)
Osteoporosis	17 (32)
Arthrosis	16 (30)
Incontinence	14 (26)
Decubitus	10 (19)
Other characteristics	
Gloomy feelings	2 (4)
Depressive feelings^c^	4 (8)
Always independent	18 (34)
Always active	10 (19)
Refuses medical examination or medical treatment	7 (13)
Recurrent falls	33 (62)
Sequence of events	39 (74)

^a^ Numbers in this category do not total 53 because most patients had more than 1 health problem.

^b^ Geriatric syndromes that occurred in at least 10 cases are presented in this table. Other medical syndromes or diseases included chronic obstructive pulmonary disease, dizziness, heart failure, constipation, and fractures.

^c^ In some of these cases, additional psychological examination was conducted because of the depressive feelings. In these cases, depression was not diagnosed.

### Geriatric Syndromes

All but 1 patient had more than 1 medical condition that caused multiple symptoms. In none of the cases were the health problems caused by a life-threatening disease. Visual impairment was the most reported symptom (34 cases [64%]), followed by hearing loss (28 cases [53%]) and chronic pain (25 cases [47%]).

### Sequence of Events and Falls as Recurrent Themes

In most cases, 2 types of circumstances were reported to be important for the patient’s wish to receive EAS. First, in 39 cases (74%), there was a sequence of events set off by an incident (the tipping point). The older patients in these cases had been dealing with multiple health problems for several years. The patients judged their suffering to be sufficient to request EAS after a decline in physical health because of the incident (eg, a fall, an infection, a hospitalization, or the loss of a close relative). Second, partly overlapping the first circumstance, in 33 cases (62%), falls and their consequences were reported. Recurrent falls caused complicated fractures in 7 cases (13%) and fear of falling in 11 cases (21%), which contributed to the experience of unbearable suffering.

### Description of Elements of Suffering

Each case summary contained a characterization of the patient’s suffering caused by MGS. These characterizations show an association between medical conditions and losses in several dimensions of life (ie, physical, psychological, social, and existential) (Table 3). In 44 cases (83%), loss of mobility was an element in the suffering of the patient. The loss of mobility ranged from not being able to go outside for a walk to being bedridden and inactive. Different kinds of fears were also an element in the experience of suffering. In addition, patients experienced social isolation and loneliness (19 [36%]). Not being able to read, watch television, or undertake meaningful activities was also an element of suffering in 19 cases (36%).

**Table 3.** Elements of Suffering^a^

Element	No. (%) of cases (N = 53)
Loss of mobility	44 (83)
Decline of mobility	16 (30)
All day sitting in a chair	12 (23)
Bedridden	9 (17)
Unable to do anything	8 (15)
Fears	21 (40)
Fear of further physical decline	20 (38)
Fear of losing independence	11 (21)
Fear of falling	11 (21)
Fear of having to move to a foster care home	10 (19)
Dependence	23 (43)
Becoming more dependent	19 (36)
Completely dependent on others	8 (15)
Social isolation	19 (36)
Loss of meaning in daily life	19 (36)
Unable to read or watch television	15 (28)
No meaningful activities	12 (23)
Loss of quality of life	9 (17)
Loss of control	5 (9)
Loss of dignity	6 (11)

^a^ Numbers do not total 53 because patients could list multiple elements of suffering.

### Conjunction of Symptoms and Events

The cases reported under the category MGS all described patients whose suffering was caused by a combination of symptoms attributable to an accumulation of syndromes. There was 1 exception, which demonstrates that a singular syndrome in combination with related experiences can be accepted by the RTEs as sufficient to meet the due care criterion of unbearable suffering without prospect of improvement.

### Practical and Procedural Aspects

All case summaries, in line with the standard procedure and the due care criteria stipulated in the Dutch law, stated that the physicians were convinced that the request was voluntary, which means that the patients made their wishes known without pressure or undue influence from others, such as family members. In addition, all published cases reflect that the physician saw no alternatives for improvement. In a number of cases, the physician had consulted a geriatric psychiatrist to rule out a reversible depression. With the exception of 1 person who received assisted suicide, all patients received euthanasia. In 32 cases (60%), a general practitioner performed the EAS; in the other 21 cases (40%), a physician from the Expertise Center Euthanasia^3^ (formerly the End-of-Life Clinic) was involved.

During the review process of 9 cases (17%), the RTEs had additional questions (25 in total) concerning the physician’s justification. Five questions were whether the patient’s unbearable suffering originated in a medically classifiable disease. The question regarding additional information at the request of the patient was asked by the RTE in 5 cases. Three times the RTE wanted additional information on possible alternatives for the EAS, and 3 times they requested information on how the physician came to be satisfied that the patient’s suffering was unbearable. Two times the RTE wanted to know more about the psychological aspect of the patient’s suffering, including the question regarding whether the patient was suffering from depression. Examples of other questions were whether consultation of an independent expert had been necessary and whether due medical care was exercised in the performance of the EAS.

After obtaining additional information from the physician who performed the EAS, the independent consultant, and other involved medical specialists, the RTEs concluded that the EAS was in accordance with the due care criteria in all but 1 case (eAppendix in the Supplement). In the case that was not approved, several due care criteria were not met. The physician was not prosecuted in court. Compared with EAS in cancer cases, cases of MGS had a greater chance of generating more questions during the review procedures of the RTEs. Physicians of the Expertise Center Euthanasia were 5 times more likely to be questioned.^4^

## Discussion

The patients who received EAS because of MGS were the oldest old. Most (77%) of the patients were women. None of them had a life-threatening condition, and all except 1 patient with a single geriatric condition had MGS, such as visual impairment and hearing loss. Pain and chronic tiredness were also common.

This study is the first, to our knowledge, to describe case reports of EAS for MGS. Two studies^11,12^ have analyzed cases of EAS for patients with psychiatric illnesses. Additional literature on the experiences concerning end-of-life decisions for the oldest old is scarce. Available studies^13,14^ reveal that fear of suffering, the wish to remain living at home, and the need for control are important elements in end-of-life decision-making. Although a medical condition associated with old age with symptoms could be determined in all 53 cases analyzed in this study, the case descriptions show that suffering occurred on multiple dimensions besides the medical one. This finding corresponds with the influential view of Cassell^15^ that the interconnectedness and the interplay among physical, psychological, social, and existential experiences are crucial for a deeper understanding of suffering.^16^ Suffering not only is a matter of pain and other physical symptoms but also has psychological, social, and existential dimensions.^15^ In addition, suffering has a temporal dimension: it can be triggered by becoming aware of what the future holds.^17^ The present analysis shows that fearing the future, fearing further physical decline, becoming more dependent, or losing control over the situation are important aspects of suffering. This finding is in line with previous research^18^ into requests for EAS by patients with end-stage cancer. In patients with MGS, these fears seem to emerge after a sequence of events. Furthermore, in 74% of the cases, an incident was reported as a decisive factor in the request for EAS. These incidents did not merely add to the accumulation of health problems. It has been observed that such incidents can be seen as a “tipping point, a warning of functional decline, dependence and isolation.”^19^^(p 904)^ In 33 of the 39 cases with incidents, this point concerned a fall that negatively affected different life dimensions. This finding confirms previous studies in which falls were interpreted as a starting point for reflection on life^20^ and a factor associated with the development of a wish to die.^21^

### Strengths and Limitations

This study has strengths and limitations. Its primary strength is its exploration of the case summaries of the RTEs in the category of MGS. These summaries describe real EAS cases and are the only accessible source to study EAS in patients suffering from MGS. Nevertheless, this study is limited by the fact that the published cases are a selection of a larger number of dossiers. For example, in 2018, the RTEs reviewed a total of 205 cases of EAS for patients with MGS. In addition, data were extracted from secondary official state documents. Such documents represent a shortened and specific version of realities, suitable for publication on an open access website^22^ and therefore containing little social history. Occasionally, a spouse or children are mentioned, but neither a person’s family structure nor living arrangement could be reconstructed.

In addition, there is a risk of underreporting cases of euthanasia. Two partly overlapping sources of underreporting exist. First, physicians sometimes misclassify their actions. Second, physicians who perform euthanasia do not always report this action to the RTEs. With regard to reporting to the RTEs, 81% of all cases of euthanasia were reported in 2015.^4^ Conclusions about the numbers and characteristics of patients with MGS among these misclassified and/or unreported cases cannot be drawn because specific data are not available.

## Conclusions

According to these findings, an accumulation of geriatric syndromes alone is insufficient to explain the unbearableness of suffering that leads to a request for EAS in older persons with MGS. In this study, all cases referred to patients who had been suffering from MGS for several years. At a certain moment in time, the suffering resulted in a request for EAS. Given that patients were already suffering from the geriatric syndromes for a long time, the findings suggest that it is not only the total number of these geriatric syndromes that is associated with unbearable suffering (and a granted request) but also the sum of these problems (often in combination with a tipping point incident) in conjunction with the patient’s medical history, life history, personality, and values that gives rise to suffering that the patient in question experiences as unbearable and without prospect of improvement. This finding also may also explain why, in some exceptional cases, the medical dimension of the suffering can also be based on only 1 geriatric syndrome that, in combination with social and existential problems associated with that syndrome, may result in unbearable suffering. In summary, in most cases, experiences in the social and existential dimensions are intertwined with the medical dimension of suffering. The variety of relevant elements in these complex cases raises the question of what the role of these different elements should be in the assessment of requests for EAS and which expertise is needed for optimal care for these older persons.

## Notes

Supplement. **eAppendix.** Review Procedures of the Dutch Regional Review Committees
