# Supplementary material for: Euthanasia and Physician-Assisted Suicide in Patients With Multiple Geriatric Syndromes
Source: JAMA Intern Med. 2020 Dec 7;181(2):1–6. doi: 10.1001/jamainternmed.2020.6895 (PMC7851730; doi:10.1001/jamainternmed.2020.6895)
Supplement: Supplement. — eAppendix. Review Procedures of the Dutch Regional Review Committees [file jamainternmed-e206895-s001.pdf]

Supplementary Online Content

van den Berg V, van Thiel G, Zomers M, et al. Euthanasia and physician-assisted suicide in patients with multiple geriatric syndromes. *JAMA Intern Med.* Published online December 7, 2020. doi:10.1001/jamainternmed.2020.6895

eAppendix. Review Procedures of the Dutch Regional Review Committees

This supplementary material has been provided by the authors to give readers additional information about their work.

© 2021 van den Berg V et al. *JAMA Internal Medicine.*

eAppendix. Review Procedures of the Dutch Regional Review Committees

The copyright owner of this infographic is the RTE. We gained official permission to use the copyrighted infographics.

Reference: https://english.euthanasiecommissie.nl/the-committees/documents/publications/euthanasia-code/euthanasia-code-2018/euthanasia-code-2018/euthanasia-code-2018

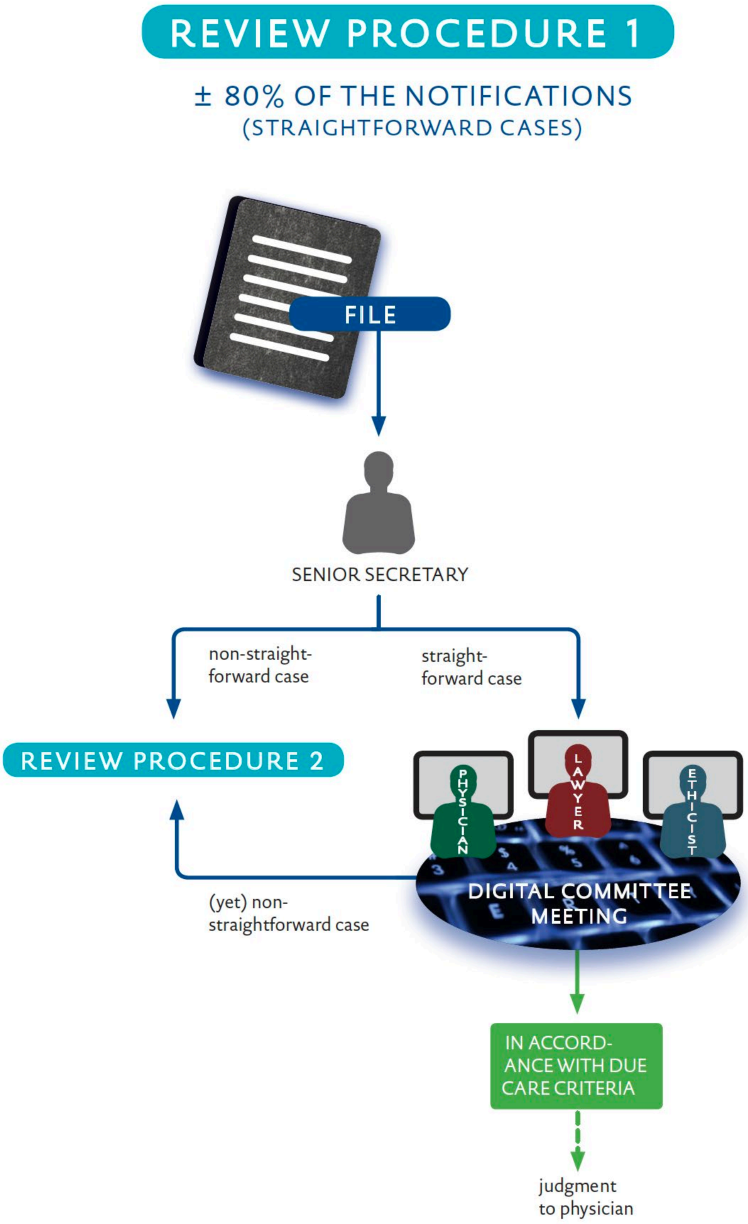

© 2021 van den Berg V et al. *JAMA Internal Medicine.*

© 2021 van den Berg V et al. *JAMA Internal Medicine.*

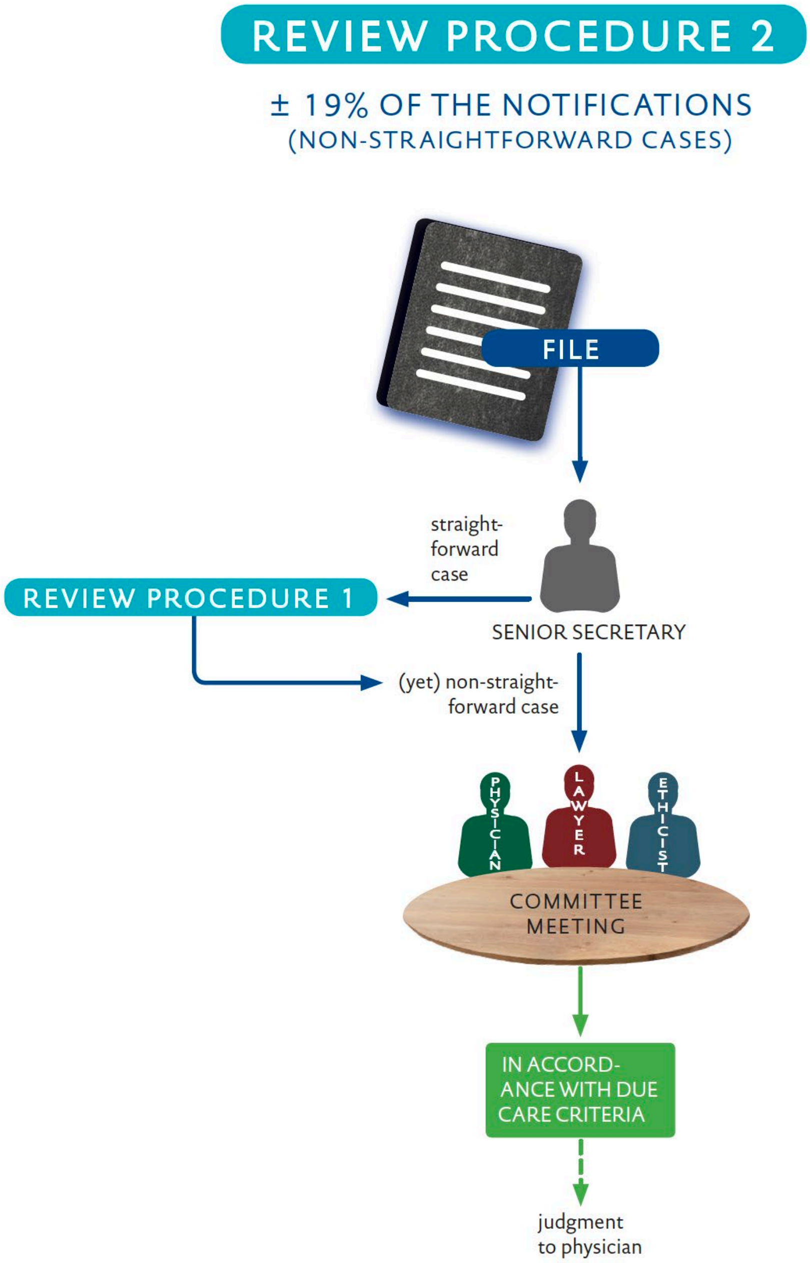

© 2021 van den Berg V et al. *JAMA Internal Medicine.*

REVIEW PROCEDURE 3

±1% OF THE NOTIFICATIONS (NON-STRAIGHTFORWARD CASES)

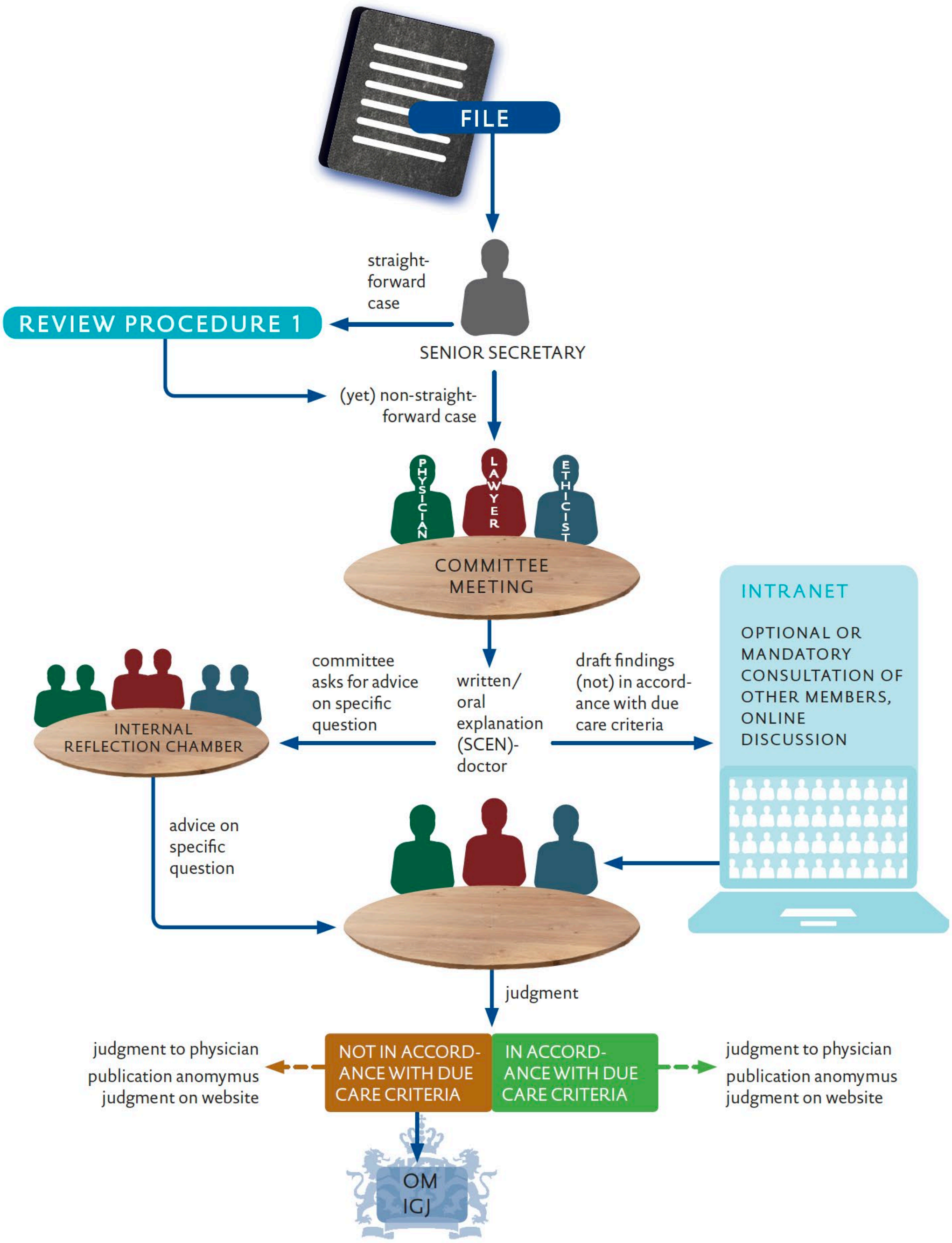

© 2021 van den Berg V et al. *JAMA Internal Medicine.*
